# Revision of the genus *Neometopina* (Hemiptera, Fulgoromorpha, Delphacidae) from China

**DOI:** 10.3897/zookeys.307.4660

**Published:** 2013-06-10

**Authors:** Xiao-hui Hou, Lin Yang, Xiang-sheng Chen

**Affiliations:** 1 The Provincial Key Laboratory for Agricultural Pest Management of Mountainous & Region/ Institute of Entomology, Guizhou University, Guiyang, 550025, China; 2 Zunyi Medical College, Zunyi, 563099, China

**Keywords:** Hemiptera, Fulgoroidea, Delphacidae, Delphacini, *Neometopina*, new combination, synonymy

## Abstract

The planthopper genus *Neometopina* Yang (Hemiptera: Fulgoroidea: Delphacidae: Delphacinae: Delphacini) is revised to include 2 species: *Neometopina penghuensis* Yang, 1989 (China: Guizhou: Maolan), and *Neometopina orientalis* (Qin & Zhang) **comb. n.** (China: Hainan: Bawangling) (transferred from *Laminatopina* Qin & Zhang). The included species are described and illustrated, and a key to species is provided.

## Introduction

The genus *Neometopina* (Hemiptera: Fulgoroidea: Delphacidae: Delphacinae: Delphacini) was erected by Yang (1989) with 1 species from Penghu island near Taiwan. Subsequently *Laminatopina*, based on the *Laminatopina orientalis* (from Hainan Province, China), was erected by Qin and Zhang (2007). Here *Laminatopina* is placed as a junior synonym of *Neometopina* Yang, with the only included species, *Laminatopina orientalis* (Qin & Zhang) is transferred as *Neometopina orientalis* (Qin & Zhang, 2007) comb. n.

A key for identifying the species of genus *Neometopina* is also provided.

## Material and methods

Morphological terminology used in this work follows that of Yang and Yang (1986). The genital segments of the examined specimens were macerated in 10% KOH and drawn from preparations in glycerin jelly aid of a light microscope. Illustrations of the specimens were made with a Leica MZ 12.5 stereomicroscope. Spinal formula means the numbers of apical spines of the hind tibiae and 1^st^ and 2^nd^ hind tarsomeres. The type specimens and examined specimens are deposited in the Insect Collection at the Institute of Entomology, Guizhou University, Guiyang, Guizhou Province, China (IEGU).

## Taxonomy

### Neometopina

Yang & Yang

http://species-id.net/wiki/Neometopina

Neometopina Yang & Yang 1989: 301. Type species: *Neometopina penghuensis* Yang & Yang, 1989, by original designation.
Laminatopina Qin & Zhang 2007: 168. Type species: *Laminatopina orientalis* Qin & Zhang, 2007. Monotypic. syn. n.

#### Diagnosis.

Species of *Neometopina* are characterized by the large size (lengths ranging from 4.62–4.82mm in macropterous males), body slender; general color pale yellowish brown with narrow brown stripes on frons and postclypeus, vertex with outer area black, median carina and two sides of pro- and mesonotum whitish yellow; forewings without spot, rounded at apex; vertex longer medially than broad at base, acutely rounding into frons; median carina of frons forked at base; antennal cylindrical, extending to the level of frontoclypeal suture; lateral carinae of pronotum not attaining hind margin; male anal segment ring-like, caudoventral margin produced medially into a large spinose process; pygofer without medioventral process, diaphragm broad with a distinct plate-like process at each side of dorsal margin; phallus tubular with one strong process arising; suspensorium with dorsal part Y-shaped, ventral part ring-like; genital styles extremely long, distinctly narrowed subapically to acute apex.

#### Description.

Head including eyes narrower than pronotum. Vertex longer than broad at base, lateral carinae straight, submedian carinae not really uniting at apex, fastigium acutely rounded, Y-shaped carina with stem existence, basal compartment wider at base than greatest length. Frons longer in middle line than wide at widest part about 3:1, widest at apex or median, with median carina forked at base. Rostrum reaching to meso-trochanters or metacoxae. Antennae cylindrical, relatively long, basal segment longer than wide, shorter than second. Pronotum with lateral carinae not attaining hind margin. Spinal formula of hind leg 5-7-4. Post-tibial spur with about 20 teeth.

Male genitalia: Anal segment ring-like, apical margin produced medially into a large spinose process. Pygofer without medioventral process. Diaphragm broad, with dorsal margin produced at median and plate-like process at each side. Phallus simple with large process at base or median. Genital styles long, simple.

#### Host plant.

Unknown.

#### Distribution.

China (Taiwan, Guizhou, Hainan).

#### Discussion.

Members of this genus are superficially similar to species of the genus *Eumetopina* Breddin, but differs in having the following combination of characters: pygofer without medioventral process but with a plate-like process at each side of dorsal margin of diaphragm; shape of phallus and frons.

*Laminatopina* Qin & Zhang (2007) is a junior synonym of *Neometopina* Yang. Features purporting to separate the genera are not generic features, but only species-level differences. For example, the stem of Y-shaped carina weak or distinct, rostrum reaching to meso-trochanters or metacoxae, and the shape and process of phallus. Secondly, based on the examined specimens, the description of the genera *Neometopina* and *Laminatopina* have some question due to the observation angle. For example, specimens of *Neometopina* basal compartment wider at base than greatest length, male anal segment with median process not produced in ventral margins, suspensorium with the dorsal portion Y-shaped, diaphragm distinctly projected dorsomedially, the slender plate-like of diaphragm processes at each side, the specimens of *Laminatopina* male genital styles diverging.

#### Key to Species of *Neometopina*

**Table:** 

1	Anal segment with median spinose process acute and slightly turned right apically, with a small process in ventral margins each side, pygofer with slender and plate-like process at each side of dorsal margin of diaphragm, phallus with large process at dorsal median side, suspensorium with dorsal part Y-shaped and tubbiness	*Neometopina penghuensis* Yang
–	Anal segment with median spinose process bifurcated apically, pygofer with broad and plate-like process at each side of dorsal margin of diaphragm, phallus with large process at base, suspensorium with dorsal part Y-shaped and slightness	*Neometopina orientalis* (Qin & Zhang) comb. n.

### Neometopina orientalis

(Qin & Zhang, 2007) comb. n.

http://species-id.net/wiki/Neometopina_orientalis

Figs 1–14

Laminatopina orientalis Qin & Zhang 2007: 168 (orig. descrip.).

#### Diagnosis.

This species is distinguished by the apical forking of anal segment process, suspensorium with dorsal part Y-shaped tenuous, phallus tubular with basal process as long as phallus, diaphragm with large and plate-like process at each side of dorsal margin.

**Description.** *Macropterous male*: Body length (from apex of vertex to the tip of forewing): male 4.62–4.82 mm; forewing length: male 3.88–4.08 mm.

*Coloration*: General color uniformly pale yellowish orange. Vertex with outerarea to submedian carinae dark brown, intercarinae of frons and postclypeus with narrow brown stripes, genae dark brown. Median carina of pro- and mesonotum whitishyellow, inner lateral carinae with light yellowish brown stripe. Ocelli dark. Eyesdark brown to black. Dorsum of abdomen yellowish orange.

*Structure*: Head including eyesnarrower than pronotum (0.88:1). Vertex longer submedially than wider at base about 1.3:1, as wide at apex as at base, lateral margins of vertex in dorsal view subparallel, submedian carinae originating from near 1/3 base of lateralcarinae, not uniting at apex, basal compartment wider at base than greatestlength. Frons longer in middle line than wide at widest part about 2.5:1, widest at level of ocelli, lateral carinae slightly sinuate, median carina forked at base. Postclypeus wider at base than frons at apex. Rostrum reaching to metacoxae. Antennae terete,reaching frontoclypeal suture, basal segment longer than wide about 1.5:1, shorter than second about 1:2.6. Lateral carinae of pronotum slightly curved. Post-tibial spur with about 20–22 black-tipped teeth. Tegmina longer than widest part about 3.6:1, rounded at apex.

*Male genitalia*: Anal segment of male large, median spinose process straight,apex bifurcated. Pygofer in profile distinctly wider ventrally than dorsally, laterodorsal angle not produced, in posterior view with opening longer than wide. Phallus tubular, apex enlarged, basal process as long as phallus, broad at basal half. Suspensorium with the dorsal portion Y-like and slender. Diaphragm broad, dorsomediallywith a distinct projection, pigmented and sclerotized, bilaterally with a large plate-like process. Opening for genitalstyles large, dorsal margin produced into a small lobe medially,ventral margin evenly curved. Genital styles extremely long, sinuate, slightlywidened subapically and greatly narrowed suddenly to acute apex, obviously concave medially.

#### Host plant.

Unknown.

#### Distribution.

South China (Hainan Province).

#### Specimens examined.

2♂♂(IEGU): CHINA: Hainan: Wuzhishan, 18°53'N, 109°41'E, 13 Apr 2009, lamping, X. X. Hou.

**Figures 1–14.**
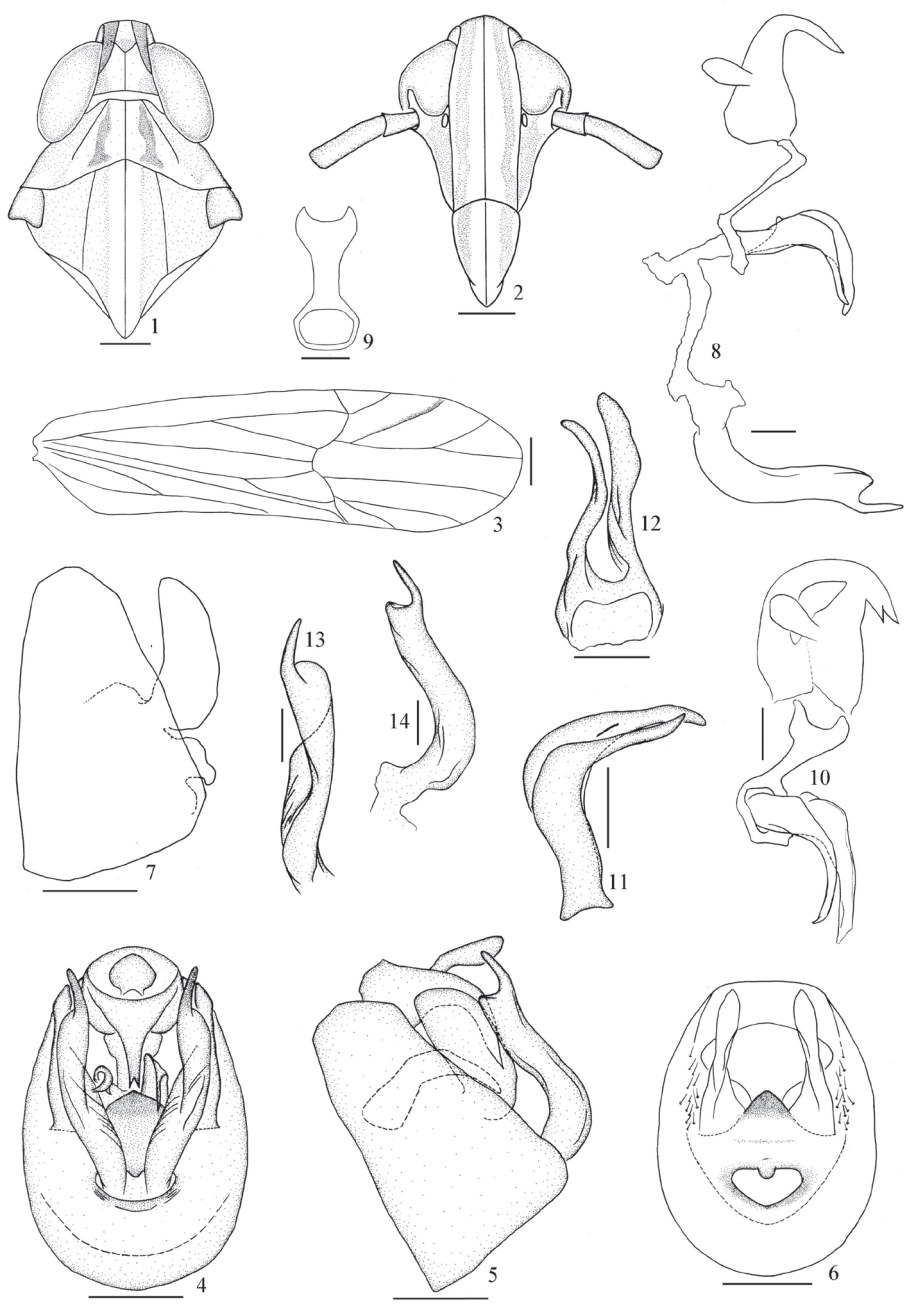
*Neometopina orientalis* (Qin & Zhang, 2007) comb. n. **1** head and thorax, dorsal view **2** fons and clypeus **3** forewing **4** male genitalia, posterior view **5** the same, lateral view **6** male pygofer, posterior view **7** the same, lateral view **8** anal segment, aedeagus and genital style, left lateral view **9** suspensorium, posterior view **10** anal segment, suspensorium and aedeagus, posterior- lateral view **11** aedeagus, lateral view **12** aedeagus, ventral view **13** genital style, posterior view **14** the same, lateral view. Scale 0.2 mm (Figures **1–7**); 0.1 mm (Figures **8–14**).

### Neometopina penghuensis

Yang

http://species-id.net/wiki/Neometopina_penghuensis

Figs 15–27

Neometopina penghuensis Yang 1989: 301 (orig. Descrip.); Ding 2006: 470.

#### Diagnosis.

This species is distinguished by the acute end of anal segment process, suspensorium with dorsal part Y-shaped stubby, phallus strongly compressed laterally with large plate-like process turning left dorsomedially, diaphragm with moderate and plate-like process at each side of dorsal margin.

#### Description.

*Macropterous*: Body length (from apex of vertex to the tip of forewing): male 4.76 mm, female 5.56 mm; forewing length: male 4.24 mm, female 4.66 mm.

*Coloration*: General color pale yellowish brown to dirty yellowish brown. Vertex with outer area to submedian carinae black brown, frons with narrow stripe, brown, genae yellowish brown. Ocelli dark. Eyesdark brown to black. Dorsum of abdomen brown.

*Structure*: Head including eyesnarrower than pronotum (0.79:1), Vertex longer submedially than wide at base about 1.3:1, as wide at apex as at base, apical margin transverse, lateral margins of vertex in dorsal view subparallel, submedian carinae originating from near 1/4 base of lateralcarinae, not uniting at apex, basal compartment wider at base than greatestlength. Frons longer in middle line than wide at widest part about 2.2:1, widest at level of ocelli, lateral carinae straight, median carina forked at base. Postclypeus large, as wide at base as frons at apex. Rostrum reaching to mesochanters. Antennae cylindrical, reaching slightly beyond frontoclypeal suture, basal segment longer than wide about 1.4:1, shorter than second about 1:2.5. Lateral carinae of pronotum curved. Post-tibial spur with about 20–23 teeth. Tegmina longer than widest part about 3.5:1, rounded at apex.

*Male genitalia*: Anal segment of male large, with a stout, twist median spinose process which pointing to the right apically, with a small process in ventral margins each side. Pygofer in profile distinctly wider ventrally than dorsally, laterodorsal angle not produced, in posterior view with opening longer than wide. Phallus strongly compressed laterally with large plate-like process turning left dorsomedially, which gradually tapering to apex. Suspensorium with the dorsal portion Y-shaped and stubby. Diaphragm broad, dorsomedially with a large process, with a pair small plate-shaped projection at each side. Opening for genital styles, with a small process at dorsal margin medially, with curved at ventral margin. Genital styles long and slender, narrowed near apex, evenly convex laterad medially.

#### Host plant.

Unknown.

#### Distribution.

South China (Guizhou and Taiwan Province).

#### Specimens examined.

6♂♂, 4♀♀(IEGU): CHINA: Guizhou: *Libo*: Maolan, 25°30'N, 108°10'E, 22 Oct 1998, X. S. Chen.

**Figures 15–27.**
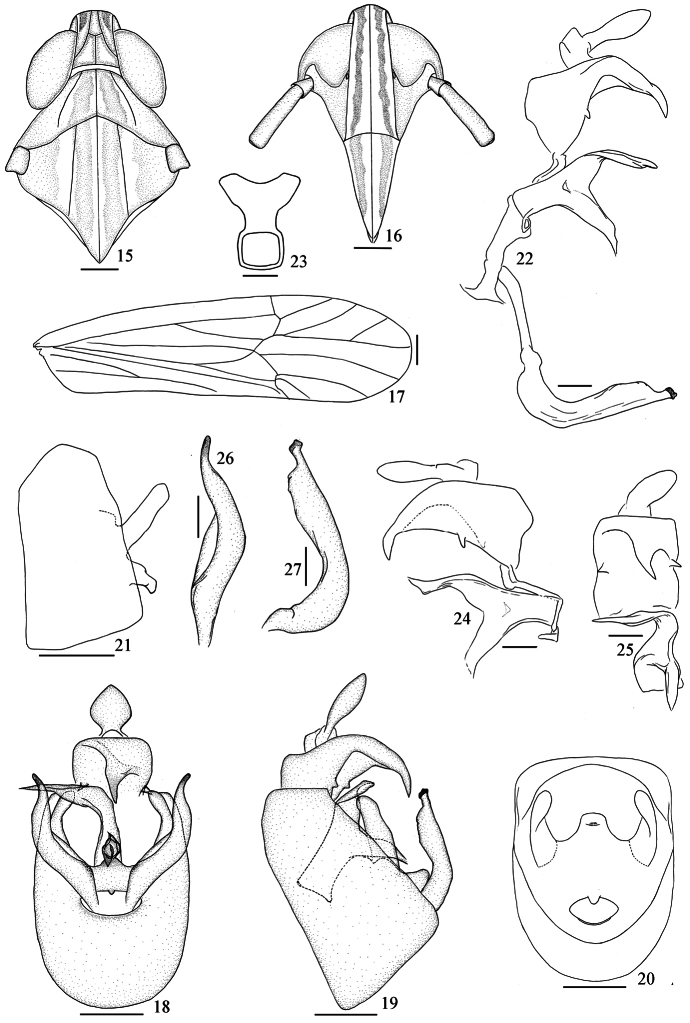
*Neometopina penghuensis* Yang, 1989 **15** head and thorax, dorsal view **16** fons and clypeus **17** forewing **18** male genitalia, posterior view **19** the same, lateral view **20** male pygofer, posterior view **21** the same, lateral view **22** anal segment, aedeagus and genital style, left lateral view **23** suspensorium, posterior view **24** anal segment, suspensorium and aedeagus, right lateral view **25** the same, posterior view **26** genital style, posterior view **27** the same, lateral view. Scale 0.2 mm (Figures **15–21**); 0.1 mm (Figures **22–27**).

## Discussion

The characteristics of genus *Laminatopina* are similar to the genus *Neometopina*, so the former as a new genus was erected by Qin and Zhang (2007). But the feature of the *Laminatopina orientalis* (such as vertex with basal compartment wider at base than greatest length; the stem of Y-shaped carina weak; rostrum reaching to metacoxae; especially by the features of the male genitalia: male anal segment with median process not produced in ventral margins; diaphragm distinctly projected dorsomedially; suspensorium with the dorsal portion Y-shaped; phallus tubular, decurved, and with one strong process arising basally; genital styles strongly diverging) are only the characteristics of species, and are correspond with the characteristics of genus *Neometopina* completely. So *the genus Laminatopina only is a* junior synonym of *Neometopina* Yang, and *the species Laminatopina orientalis* (Qin & Zhang) is transferred to the genus *Neometopina* as a new combination (*Neometopina orientalis* (Qin & Zhang, 2007) comb. n.).

## Supplementary Material

XML Treatment for Neometopina

XML Treatment for Neometopina orientalis

XML Treatment for Neometopina penghuensis
